# Therapeutical Effect of Alcohol

**Published:** 1872-12

**Authors:** 


The Therapeutical Effect of Alcohol has lately been the subject of repeated investigations in Germany. Professor Binz, of Bonn, delivered a lecture before the Society of Natural Sciences and Medicine of the Lower Rhine, on the 4th of December last, on this subject; and his pupil, C. Bouvier, in an essay under the title, "Pharmacological studies on the effect of Alcohol," just published by A. Hirschwald, at Berlin, subjects the leading ideas of his teacher to further experimental investigations. He proves beyond a doubt that alcohol decreases the temperature of the body, when it has not been habitually used, long before drunkenness, much less intoxication, is established, and that this effect is the consequence of its retarding influence on the metamorphosis of tissue, and the chemical process which produces the animal heat.—*Berliner Klinische Wochenschrift, No.* 37, 1872. R.

This discovery places the use of alcohol in low graded or adynamic fevers, for instance, typhoid fevers, on a very different physiological basis to that which it so far has generally occupied in the mind of medical practitioners. It has been administered as a so-called stimulant, for the purpose of lessening the frequency and increasing the force and volume of the pulse, and quieting the delirium and nervous agitation of the patient, under the supposition that it had a specific power to increase the nerve force of the brain. It now appears that this so-called stimulating effect mnst be owing to a diminution of that morbid combustion and disintegration of tissue and nitrogenous material, which, by its involving the substance of the brain and nervous system, generally seems to be the principal cause of that characteristic train of nervous symptoms so frequently observed in these fevers. R.

